# A Prospective Cohort Study of the Effects of Adjuvant Breast Cancer Chemotherapy on Taste Function, Food Liking, Appetite and Associated Nutritional Outcomes

**DOI:** 10.1371/journal.pone.0103512

**Published:** 2014-07-31

**Authors:** Anna Boltong, Sanchia Aranda, Russell Keast, Rochelle Wynne, Prudence A. Francis, Jacqueline Chirgwin, Karla Gough

**Affiliations:** 1 Cancer Council Victoria, Melbourne, Australia; 2 Cancer Institute NSW, Eveleigh, NSW, Australia; 3 Department of Cancer Experiences Research, Peter MacCallum Cancer Centre, East Melbourne, Victoria, Australia; 4 Melbourne School of Health Sciences, The University of Melbourne, Carlton, Victoria, Australia; 5 Centre for Physical Activity and Nutrition Research, Deakin University, Burwood, Victoria, Australia; 6 Breast Medical Oncology, Peter MacCallum Cancer Centre, East Melbourne, Victoria, Australia; 7 Breast Medical Oncology, Eastern Health, Australia; Davidoff Center, Israel

## Abstract

**Background:**

‘Taste’ changes are commonly reported during chemotherapy. It is unclear to what extent this relates to actual changes in taste function or to changes in appetite and food liking and how these changes affect dietary intake and nutritional status.

**Patients and methods:**

This prospective, repeated measures cohort study recruited participants from three oncology clinics. Women (*n* = 52) prescribed adjuvant chemotherapy underwent standardised testing of taste perception, appetite and food liking at six time points to measure change from baseline. Associations between taste and hedonic changes and nutritional outcomes were examined.

**Results:**

Taste function was significantly reduced early in chemotherapy cycles (p<0.05) but showed recovery by late in the cycle. Ability to correctly identify salty, sour and umami tastants was reduced. Liking of sweet food decreased early and mid-cycle (*p*<0.01) but not late cycle. Liking of savory food was not significantly affected. Appetite decreased early in the cycle (*p*<0.001). Reduced taste function was associated with lowest kilojoule intake (*r* = 0.31; *p* = 0.008) as was appetite loss with reduced kilojoule (*r* = 0.34; *p* = 0.002) and protein intake (*r* = 0.36; *p* = 0.001) early in the third chemotherapy cycle. Decreased appetite early in the third and final chemotherapy cycles was associated with a decline in BMI (*p* = <0.0005) over the study period. Resolution of taste function, food liking and appetite was observed 8 weeks after chemotherapy completion. There was no association between taste change and dry mouth, oral mucositis or nausea.

**Conclusion:**

The results reveal, for the first time, the cyclical yet transient effects of adjuvant chemotherapy on taste function and the link between taste and hedonic changes, dietary intake and nutritional outcomes. The results should be used to inform reliable pre-chemotherapy education.

## Introduction

Taste is one of the five senses and interacts with smell, touch and other physiological cues to affect the wider perception of *flavor*. Taste function is defined as the perception derived when chemical molecules stimulate taste receptor fields in areas of the tongue, soft palate and oropharyngeal region of the oral cavity to perceive the five basic taste qualities (sweet, sour, salty, bitter and umami) [1], measured via standardised processes [2]. Food hedonics, which also contributes to flavour perception, encompasses food liking: the immediate experience or anticipation of pleasure from the oro-sensory stimulation of eating a food [3], and appetite: a psychobiologically based sensation related to the maintenance of eating and a desire for specific foods [4].

Chemotherapy is known to affect other senses with ototoxicity and peripheral neuropathy recognized treatment-related toxicities, which in some cases may be permanent [5], [6]. ‘Taste’ changes are commonly reported by people receiving chemotherapy [7] even among those who do not report nausea. It is unclear to what extent this relates to altered taste function per se or to changes to the sense of smell or touch (including oral dryness) or to hedonic aspects such as food liking, or appetite, also described colloquially by patients and clinicians as ‘taste’ [8].

‘Taste’ changes in oncology populations have been linked to adverse effects on quality of life, morbidity and mortality due to an association with inadequate energy and nutrient intake, weight loss, malnutrition [11], reduced compliance with treatment regimens [9], reduced immunity [10], [11], altered food relationships [12], changed food rituals [13], emotional distress and interference with daily life [14]. The extent to which true taste problems play a role in these scenarios is unknown. The ability to perceive taste sensations guides food choice, which in itself is a determinant of health [15]. Because changes in taste function, liking of food and appetite all have the potential to underpin changes in dietary intake and nutritional status, understanding the extent of the contribution of each would help inform the development of effective interventions in future.

### Study Objectives

The primary objective of this study was to measure the effect of adjuvant breast cancer chemotherapy on taste function and food hedonics across the treatment trajectory. The primary hypothesis was that taste function and food hedonics would be adversely affected by chemotherapy and that the greatest changes to taste function and food hedonics would occur early in a chemotherapy cycle. It was also hypothesised that changes in taste function and food hedonics would be associated with alterations in dietary intake and nutritional status. A secondary objective was to assess the relationship between changes in taste function and toxicities.

## Methods

### Study Design

This was a prospective, multi-centre cohort study that recruited patients planned for adjuvant chemotherapy for breast cancer at three hospital-based oncology clinics in Melbourne, Australia from April to December 2011. Potentially eligible patients were identified via medical oncology clinics and breast cancer multidisciplinary meetings.

### Ethics statement

Institutional ethics approval was granted at Eastern Health and Peter MacCallum Cancer Centre and written informed consent was obtained from each patient before enrolment.

### Participants

Patients aged 18 years or over scheduled to receive an anthracycline and/or taxane containing chemotherapy regimen for the adjuvant treatment of resected invasive breast cancer were eligible to participate. Patients were required to be able to read and converse in English. Exclusion criteria included chemotherapy already initiated, concurrent radiotherapy, previous radiotherapy to the head and neck region, or presence of cognitive impairment that might impact study outcomes.

### Variables

**Figure 1:**
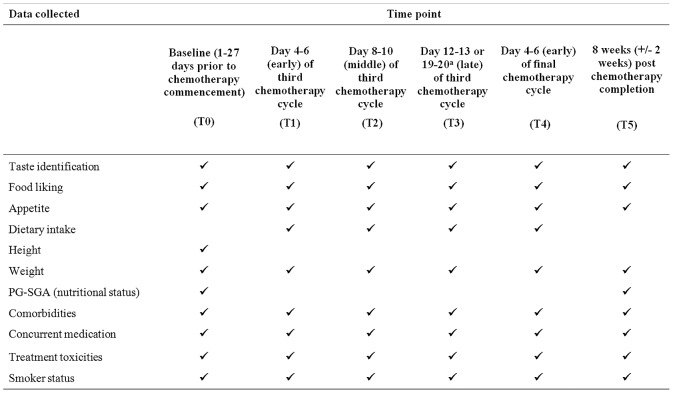
Summary of outcome data collected at each study time point. ^a^Day 12–13 of the third chemotherapy cycle if receiving 14 day chemotherapy cycles. Cycle 3, Day 19–20 if receiving 21 day chemotherapy cycles.

Outcome measures were number of tastants identified correctly, food liking score, appetite rating, daily energy (kJ) and macronutrient (protein, fat and carbohydrate) intake, weight, body mass index (BMI) and nutritional status (Figure 1). Demographic and clinical data collected were obtained for age, years of education, smoking status, BMI, concurrent medications, presence of conditions implicated in taste function (liver or renal dysfunction, sinusitis, diabetes) and treatment related toxicities (nausea, dry mouth and oral mucositis).

### Data sources and measurement

Figure 1 details study assessment time points and data collected. Time points were selected in order to avoid days in the chemotherapy cycle when patients may be taking corticosteroids. Intra-cycle time points were chosen to assess effects early, middle and late in a cycle. Stage of chemotherapy treatment was selected based on a qualitative study with chemotherapy recipients who reported symptoms being apparent by the third chemotherapy cycle and resolving by 6–8 weeks after completion of chemotherapy treatment [16].

Demographic and clinical data were obtained from the patients’ medical records and via direct questioning of the participant. Clinical assessment of relevant chemotherapy toxicities was performed at each study appointment in accordance with the US Department of Health and Human Services Common Terminology Criteria for Adverse Events (CTCAE) v.4.03, 2010.

Taste identification testing was performed in accordance with the International Standards Organization (ISO), ISO 3972∶2011-Sensory Analysis-Methodology-Method of Investigating Sensitivity of Taste as part of a taste identification task. Tastants and their corresponding concentrations were: sucrose 300 mM, NaCl 200 mM, citric acid 5 mM, caffeine 10 mM, MSG 200 mM. These solutions were prepared in the Deakin University sensory laboratory from food grade chemicals and deionised water in the 7 day period prior to testing. At testing, five 2 ml solutions corresponding to the five basic taste qualities (sweet, salty, sour, bitter and umami) were each tasted in a single ‘sip and spit’ technique to determine the total number and individual tastants identified correctly at each time point. The mouth was rinsed with room temperature purified water three times before and after sampling and expectorating each solution. Perceived taste quality was identified by selecting one of seven choices. Correct responses were *sweet* for sucrose, *salty* for NaCl, *sour* for citric acid, *bitter* for caffeine, and *savoury* for MSG. Additional choices were *none* or *metallic*. Taste identification score was assigned as 0–5 correct choices.

Food liking was assessed using a 9-point hedonic scale [17] to measure liking of a standard sweet (chocolate) and umami (soup) food item from *Like extremely* (9) to *Dislike extremely* (1). Appetite was rated on a 10-point scale from *Best appetite* (10) to *Worst appetite* (1). Before all taste tests, participants were asked to refrain from smoking, chewing gum, using toothpastes or other oral care products, or eating or drinking anything other than water for a minimum of one hour.

Dietary intake data for the preceding 24 hour period was collected by a dietitian via telephone according to the United States Department of Agriculture (USDA) automated multiple-pass approach (AMPA) [18]. Dietary data were analysed using FoodWorks 2007 (Xyris software, Queensland, Australia) and daily nutrient intake was quantified as kilojoules and grams of protein per kg of body weight and carbohydrate and fat as a proportion of daily energy intake. Investigator assisted height and self-reported weight were used to calculate BMI (kg/m^2^). The Patient-Generated Subjective Global Assessment (PG-SGA) [19] is a validated method of assessing and classifying nutritional status in oncology populations and was used at baseline (T0) and 8 weeks after completion of chemotherapy (T6) [20].

### Sample size

Sample size requirements for this study were determined based on estimates available for the hedonic scale and a difference between baseline and final follow-up (where attrition would be highest). In this case, sample size calculations were based on a paired-samples t-test with an alpha level of 0.05, 80% power, a difference of 0.8 points in food liking and a standard deviation of 2.1 [17] (a standardised difference of 0.42). Given these specifications, a total sample of 47 patients was required at final follow-up. Assuming attrition of up to 10%, a minimum of 52 patients were needed at baseline.

### Statistical analysis

Recruitment bias was assessed by comparing the age, treatment centre and cancer stage of patients who consented to participate and those who declined participation using t-tests and chi-squared (or Fisher’s exact) tests as appropriate. Analysis of food liking and appetite was carried out by fitting a linear mixed model to each outcome separately; a reference cell model was used to generate estimates of baseline means and differences between baseline and follow-up assessments with 95% confidence intervals and tests of significance. An unstructured covariance type was used to model the covariance structure among repeated measures and all models were estimated by maximum likelihood. McNemar’s test was used to assess differences between proportions of participants correctly identifying all five tastants at follow-up assessments compared with baseline. Differences were also assessed for each tastant individually. An SPSS macro created by Garcia-Granero was used to perform this test. [21] Confidence intervals generated by this macro are based on methods developed by Newcombe [22]. Kendall’s Tau-b was used to examine associations of change scores for number of tastants correctly identified and liking of sweet and savoury test food items with change in BMI and PG-SGA score. Frequency statistics were used to summarise treatment toxicities at each time point and Kendall’s Tau-b was used to examine associations of change scores for number of tastants correctly identified with treatment toxicities at corresponding follow-up assessments. Correlation coefficients were interpreted as follows: 0.1, small association; 0.3, medium association; and 0.5, large association [23]. SPSS Windows Version 21 (Chicago, IL, USA) was used for all analyses. No adjustments were made for multiplicity.

## Results

**Figure 2:**
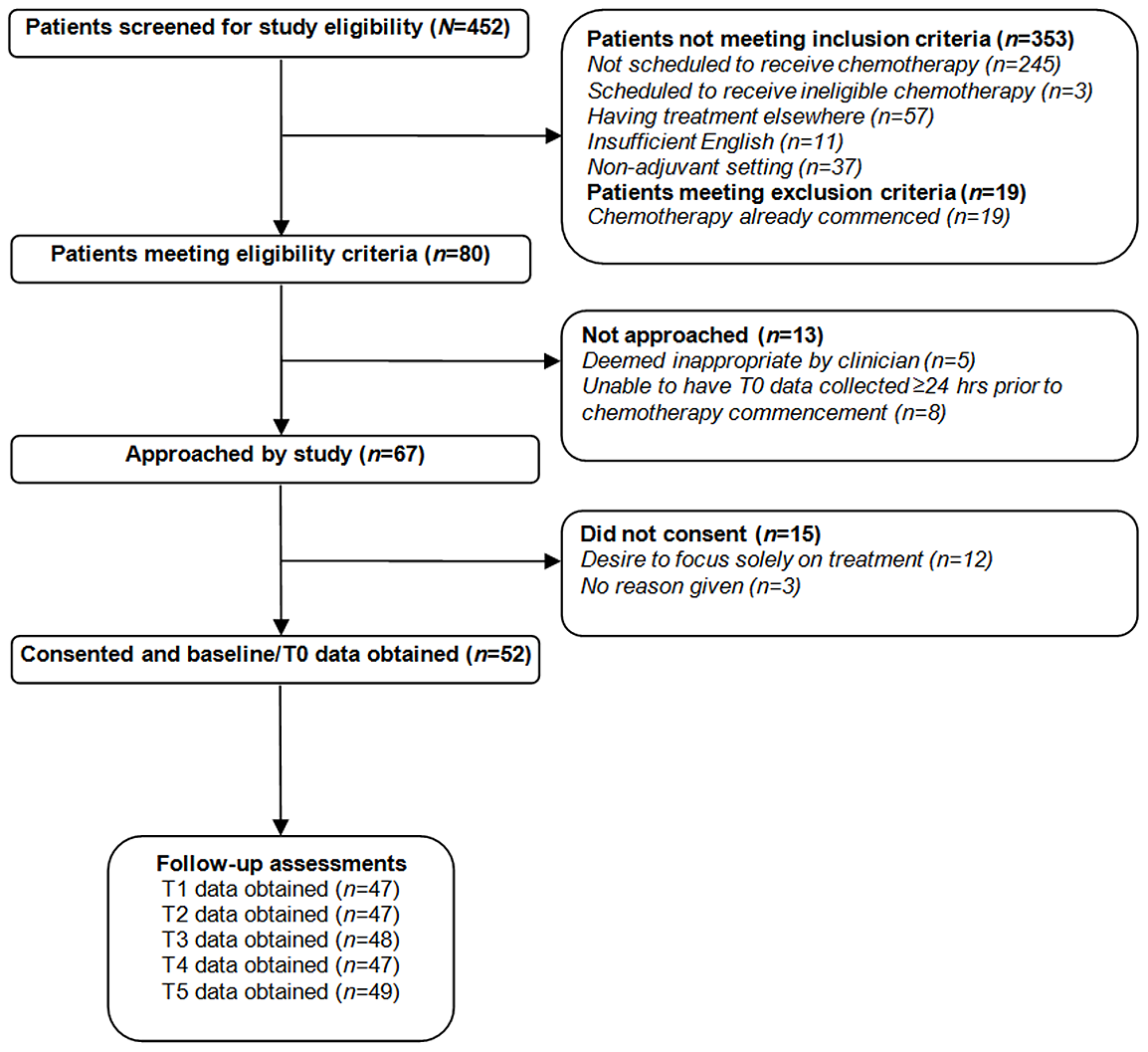
CONSORT diagram reporting numbers of individuals at each stage of the study.

Fifty-two participants were enrolled in the study. Figure 2 summarises numbers of participants screened, approached and recruited.

There were no differences between patients who consented to participate and those who declined participation in terms of age, treatment centre or stage of disease (all *p*>0.05). Compliance with data collection was high at ≥96% for clinical variables, ≥94% for demographic variables and ≥92% for all outcome measures. Demographic, clinical and social characteristics of the sample are shown in Table 1.

**Table 1:** Demographic, clinical and social characteristics of study participants and non-participants at baseline.

Characteristic	Study sample	Non-participants	*p* value^a^
**Gender**			
Female *n* **(%)**	*52* (100)	*15* (100)	
**Age**			0.35
Range	32–74	33–73	
Mean	50.4	53.3	
SD	9.7	11.6	
**Treatment Centre**			0.78
Clinic A	25	9	
Clinic B	9	1	
Clinic C	18	5	
**Schooling years**			
Range	7–21		
Mean	14.2		
SD	3.2		
**BMI**			
IQR range	24.2–31.3		
Median	26.9		
**Smoking status ** ***n*** ** (%)**			
Never smoked	24 (46)		
Ex-smoker	24 (46)		
Current smoker	4 (8)		
**Cancer stage ** ***n*** ** (%)**			0.53
I	*13* (25)	*3* (20)	
II	*23* (44)	*5* (33)	
III	*16* (31)	*7* (47)	
**Scheduled chemotherapy treatment ** ***n*** ** (%)**			
bTaxane based regimens	*24* (46)		
cAnthracyline → Docetaxel	*17* (33)		
cAnthracyline → Paclitaxel	*11* (21)		

a for comparison of responders and non-responders;

b Taxane based regimens were: TC(4): Docetaxel 75 mg/m^2^ plus cyclophosphamide 600 mg/m^2^ every 3 weeks×4 cycles: *n* = 20 patients (38.5%); or 6 cycles: TC(6), *n* = 1 (1.9%); TCarbo: Docetaxel 75 mg/m^2^ plus carboplatin AUC 6 every 3 weeks×6 cycles: *n* = 3 (5.8%);

c Sequential anthracycline → taxane regimens were: AC(4)-T(4): Doxorubicin 60 mg/m^2^ plus cyclophosphamide 600 mg/m^2^ every 3 weeks×4 cycles followed by docetaxel 100 mg/m^2^ every 3 weeks×4 cycles: *n* = 4 (7.7%); *dd*AC(4)-T(4): Doxorubicin 60 mg/m^2^ plus cyclophosphamide 600 mg/m^2^ every 2 weeks×4 cycles followed by paclitaxel 175 mg/m^2^ every 2 weeks×4 cycles (with G-CSF during each of 8 cycles): *n* = 5 (9.6%); AC(4)-T(12): Doxorubicin 60 mg/m^2^ plus cyclophosphamide 600 mg/m^2^ every 3 weeks×4 cycles followed by paclitaxel 80 mg/m^2^ weekly×12 cycles: *n* = 2 (3.8%); *dd*AC(4)-T(12): Doxorubicin 60 mg/m^2^ plus cyclophosphamide 600 mg/m^2^ every 2 weeks by 4 cycles (with G-CSF during each of 4 cycles) followed by paclitaxel 80 mg/m^2^ weekly×12 cycles: *n* = 4 (7.7%); FEC(3)-D(3): 5-fluorouracil 500 mg/m^2^ plus epirubicin 100 mg/m^2^ plus cyclophosphamide 500 mg/m^2^ every 3 weeks followed by docetaxel 100 mg/m^2^ every 3 weeks×3 cycles*: n* = 13 (25.0%).

*Notes.* Cancer stage as per AJCC Cancer Staging Manual. Seventh edition. Numbers in parentheses are total chemotherapy cycles received for each type. *dd = *dose dense (given over a 2-week cycle).

### Change in taste function

Before adjuvant chemotherapy, 33% of participants correctly identified all five tastants (Table 2). Taste function was reduced with significantly fewer participants correctly identifying all five tastants early cycle 3 (difference −18%, 95% CI −33, −3; *p* = 0.043) and early final cycle (difference −20%; 95% CI −35, −3; *p* = 0.039) compared with baseline. Fewer participants correctly identified all five tastants mid cycle 3 (difference −13%, 95% CI −28, 2; *p* = 0.15) but this difference was not significant and any effect had predominantly resolved by late cycle 3 (difference −4%, 95% CI −20, 12; *p* = 0.79). There was no difference between the proportions of participants who correctly identified all five tastants pre-chemotherapy and 2 months post-chemotherapy (difference 0%, 95% CI −15, 15; *p* = 0.77).

**Table 2:** Correct identification of standardised tastants: proportions of sample identifying all five correct at baseline and differences in proportion at subsequent time points (*N* = 52).

	^a^T0: Pre-chemo	bT1: Early cycle 3		bT2: Mid cycle 3		bT3: Late cycle 3		bT4: Early final cycle		bT5∶2 months post-chemo	
	(baseline)	Difference	*p*	Difference	*p*	Difference	*p*	Difference	*p*	Difference	*p*
**All tastants**	33	−**18 [**−**33,** −**3]**	0.043	−13 [−28, 2]	0.15	−4 [−20, 12]	0.79	−**20 [**−**35,** −**3]**	0.039	0 [−15, 15]	0.77
**Sweet**	98	0 [−8, 8]	1.00	−4 [−14, 4]	0.48	−4 [−14, 4]	0.48	−2 [−11, 6]	1.00	0 [−7, 7]	1.00
**Salty**	87	−13 [−29, 3]	0.181	−**20 [**−**35,** −**3]**	0.039	−4 [−19, 10]	0.77	−**23 [**−**39,** −**6]**	0.022	−4 [−15, 6]	0.62
**Sour**	77	−20 [−36, −2]	0.052	−19 [−36, −1]	0.067	−23 [−42, −2]	0.070	−**27 [**−**41,** −**11]**	0.004	−18 [−34, −2]	0.052
**Bitter**	52	−16 [−32, 2]	0.15	−6 [−22, 10]	0.61	−2 [−16, 12]	1.00	−13 [−29, 5]	0.24	−2 [−16, 12]	1.00
**Umami**	79	−**16 [**−**29,** −**2]**	0.046	−15 [−30, 0]	0.10	−6 [−17, 4]	0.37	−9 [−23, 6]	0.39	0 [−11, 11]	0.68

*Notes*. ^a^Pre-chemo data are percentage correct.

b All other data are differences in percentages correct (follow-up assessment minus baseline assessment) with 95% CI. All *p* are continuity-corrected.

At baseline and at all subsequent time points, sucrose as sweet and caffeine as bitter were the most and least accurately identified tastants respectively (Table 2). Further, compared with baseline, there were no differences between the proportions of participants who correctly identified either of these tastants at any follow-up time point (all *p*>0.05). Conversely, there was a significant reduction in the proportions of participants who correctly identified MSG as savoury early cycle 3 (difference −16%, 95% CI −29, −2; *p* = 0.046) and NaCl as salty mid cycle 3 (difference −20%, 95% CI −35, −3; *p* = 0.039) and early final cycle (difference −23%, 95% CI −39, −6; *p* = 0.022). Compared with baseline, fewer participants correctly identified citric acid as sour at all subsequent time points (Table 2) but only the difference between early final cycle and baseline reached significance (difference −27%, 95% CI −41, −11; *p* = 0.004).

### Change in food hedonics

Food liking and appetite (Table 3) exhibited similar patterns of differences as taste function indexed by total number of tastants correctly identified. Compared with baseline, appetite was significantly lower early cycle 3 (difference 2.1, 95% CI −2.9, −1.3; *p*<0.0005) and early final cycle (difference 2.1, 95% CI −3.0, −1.2; *p*<0.0005). Liking of sweet food was also significantly lower early cycle 3 (difference 0.9, 95% CI −1.5, −0.4; *p* = 0.002) and early final cycle (difference 1.1, 95% CI −1.7, −0.5; *p* = 0.001), as well as mid cycle 3 (difference 0.8, 95% CI −1.4, −0.3; *p* = 0.003), compared with before chemotherapy. Compared with baseline, none of the differences in liking of savoury food at subsequent time points were significant (all *p*>0.05).

**Table 3:** Food hedonics: estimates of food liking and appetite at baseline and differences at subsequent time points (*N* = 52).

	^a^T0: Pre-chemo	bT1: Early cycle 3		bT2: Mid cycle 3		bT3: Late cycle 3		bT4: Early final cycle		bT5∶2 months post-chemo	
	(baseline)	Difference	*p*	Difference	*p*	Difference	*p*	Difference	*p*	Difference	*p*
**Liking of sweet food**	7.8 (0.2)	−**0.9 [**−**1.5,** −**0.4]**	0.002	−**0.8 [**−**1.4,** −**0.3]**	0.003	−0.3 [−0.7, 0.1]	0.16	−**1.1 [**−**1.7,** −**.5]**	0.001	−0.1 [−0.4, −0.2]	0.70
**Liking of savoury food**	5.5 (0.3)	−0.5 [−1.1, 0.1]	0.085	−0.4 [−1.0, 0.2]	0.19	0.0 [−0.6, 0.6]	0.97	−0.4 [1.0, 0.2]	0.23	−0.1 [−0.7, 0.4]	0.65
**Appetite**	7.8 (0.3)	−**2.1 [**−**2.9,** −**1.3]**	<0.0005	−0.7 [−1.5, 0.1]	0.089	0.3 [−0.4, 0.9]	0.45	−**2.1 [**−**3.0,** −**1.2]**	<0.0005	0.2 [−0.5, 0.85]	0.58

*Notes*. ^a^Pre-chemo data are mean scores at baseline with the standard error of estimate in brackets.

b All other data are within-group changes in mean scores (follow-up assessment minus pre-chemo assessment) with 95% CI at specified time point.

### Association between changes in taste function and appetite and nutritional outcomes

#### Changes in taste function

There was a significant, medium-sized association between a change in taste function and kilojoule intake early cycle 3 (Tau-b = 0.31, *p* = 0.008). Participants whose ability to correctly identify all five tastants deteriorated consumed fewer kilojoules per kilogram (Table 4).

**Table 4:** Associations with taste and appetite changes from baseline to assessment points early in the third and final chemotherapy cycles.

	Taste change from baseline				Appetite change from baseline			
	T1: Early cycle 3		T4: Early final cycle		T1: Early cycle 3		T4: Early final cycle	
	Tau-b	*p* value	Tau-b	*p* value	Tau-b	*p* value	Tau-b	*p* value
**kJ per kg consumed**	**0.31**	0.008	0.10	0.37	**0.34**	0.002	0.14	0.19
**Protein (g) per kg consumed**	0.17	0.13	0.06	0.62	**0.36**	0.001	0.13	0.23
**Proportion of daily energy intake as fat**	0.07	0.54	−0.08	0.45	0.15	0.16	0.03	0.75
**Proportion of daily energy intake as CHO**	0.12	0.31	0.12	0.28	−0.13	0.22	−0.06	0.55
**Change in PG**−**SGA across study period**	−0.07	0.56	0.02	0.88	−0.11	0.33	0.18	0.092
**BMI change**	−0.01	0.91	0.12	0.28	**0.31**	0.004	**0.42**	<0.0005

*Notes.* CHO = Carbohydrate. BMI change calculated for the relevant taste/appetite change period. Medium-sized associations in bold for emphasis.

#### Changes in appetite

There were significant, medium-sized associations between reduced appetite early cycle 3 and reduced kilojoule and protein intake (Tau-b = 0.34, *p* = 0.002; and Tau-b = 0.36, *p* = 0.001, respectively) and decline in BMI early cycle 3 compared to baseline (Tau-b = 0.31, *p* = 0.004). There was also a significant, medium-sized association between changes in reduced appetite and decline in BMI early final cycle compared to baseline (Tau-b = 0.42, *p*<0.0005) (Table 4).

### Association between changes in taste, chemotherapy type and nutrition related toxicities

The majority of participants reported no symptoms of dry mouth, oral mucositis or nausea at all time points assessed. Nonetheless, there was a notable increase in the percentages of patients reporting treatment-related toxicities early and mid cycle 3 and early final cycle. None of the associations between changes in taste function at follow-up time points from before chemotherapy and self-reported toxicities were significant (all *p*>.05, Table 5).

**Table 5:** Presence of mouth dryness, oral mucositis and nausea at each time point and association between each toxicity and change in taste function.

	T0: Pre-chemo(baseline)	T1: Earlycycle 3	T2: Midcycle 3	T3: Latecycle 3	T4: Earlyfinal cycle	T5∶2 monthspost-chemo
**Mouth dryness**						
Grade 0	92.3	55.6	63.8	81.3	63.8	87.8
Grade 1	7.7	40.0	29.8	16.7	29.8	12.2
Grade 2	0.0	4.4	6.4	2.1	6.4	0.0
Correlation with change in taste function		−0.052	−0.019	−0.078	0.059	0.043
*p* value		0.70	0.88	0.55	0.65	0.76
**Oral mucositis**						
Grade 0	96.2	88.9	85.1	87.5	83.0	93.9
Grade 1	3.8	6.7	10.6	10.4	10.6	6.1
Grade 2		4.4	2.1	2.1	4.3	
Grade 3			2.1		2.1	
Correlation with change in taste function		−0.018	0.072	−0.12	−0.063	−0.029
*p* value		0.89	0.59	0.35	0.63	0.83
**Nausea**						
Grade 0	96.2	57.8	73.9	89.4	76.6	91.8
Grade 1	1.9	13.3	10.9	6.4	6.4	4.1
Grade 2	1.9	28.9	15.2	4.3	17.0	4.1
Correlation with change in taste function		0.003	−0.11	−0.091	−0.096	−0.10
*p* value		0.98	0.40	0.49	0.46	0.46

*Notes*. Data on presence of mouth dryness, oral mucositis and nausea are percentages.

## Discussion

The results of this study investigating women receiving adjuvant chemotherapy support the hypothesis that taste function is adversely affected by chemotherapy and that chemotherapy-related effects are greatest early in a chemotherapy cycle. Further, changes in taste function are cyclical and transient, as are changes in food liking and appetite. The hypothesis that chemotherapy related taste and hedonic changes are associated with alterations in dietary intake and weight was also supported, although these effects are experienced variably. Associations between change in taste function and chemotherapy-related nausea, dry mouth and mucositis, were typically small or trivial in size. Thus this study contributes new knowledge in the area of chemotherapy-related changes in taste function, food hedonics and nutritional outcomes for women receiving adjuvant chemotherapy for breast cancer.

This is the first published study to have examined taste function more than once within a single chemotherapy cycle in a sample size greater than 10 participants and the first to assess perception of all five basic taste qualities in an adult chemotherapy population. Taste assessment in previous studies of chemotherapy populations was conducted on the day of chemotherapy administration (late cycle) when patients report symptoms are at their mildest. This flaw in methodology incorrectly suggested that taste function may be unaffected by chemotherapy [24]. Data from the current study supports a recommendation that future research incorporates early-mid cycle taste measurements in its design. It has been shown previously that chemotherapy related taste changes resolve some time between the end of treatment and 3 months after [25]. This study provides new evidence that for breast cancer patients, taste problems experienced in the first 4–6 days after chemotherapy administration will likely resolve over the course of a single chemotherapy cycle, repeat with each cycle and resolve completely in as little as two months after chemotherapy completion. These findings equip clinicians with accurate information to provide breast cancer patients regarding expected nature and duration of symptoms.

Given that patients undergoing chemotherapy commonly report a reversal in preference for sweet or savoury foods and aversions to items such as chocolate and coffee [26], the difference between change in liking of the prototypical sweet food and prototypical savoury food is noteworthy. It is not known whether a change in taste function per se is responsible for the changes in liking of food observed or whether this is driven more by changes in appetite or as a result of other factors such as chemotherapy induced nausea. It is postulated that the absence of a demonstrated decrease in ability to identify sweet and bitter tastants may account for this observation, suggesting these sweet and bitter taste qualities are disproportionally (and aversively) perceptible over others. It has been shown previously that hedonic scores for sucrose solutions decrease at high concentrations in patients with poorest appetites [27] suggesting that those with poorest appetites have greatest aversion to intensely sweet items.

This study adds to limited existing evidence for the link between taste and hedonic changes, dietary behaviour and nutritional outcomes. Although a reduction in taste function was associated with a lower kJ intake, appetite loss was more strongly related to dietary inadequacy and weight loss than was change in taste sensitivity. Reduced appetite had a medium-sized association with reduced BMI and a small-sized association with worsening nutritional status, however not all participants suffering taste or appetite deficits lost weight. The relationship between taste and food hedonics and alterations in dietary quality and nutritional outcomes was specific to early breast cancer populations in this study and should be tested in other clinical scenarios not least because of bidirectional weight change and its variable clinical implications.

Varying use of anti-nausea medications may be a confounding factor in this study as nausea has previously been associated with learned food aversions during chemotherapy treatment [28]. It is postulated that degree of nausea control is likely to influence self-rated appetite and food liking. Future studies should consider incorporating a standardised anti-emetic regimen. Previous studies of taste and food hedonics in chemotherapy populations suffered from methodological issues of heterogeneity in cancer type, stage and prescribed chemotherapy. This was not a factor in the current study. However, time lapse between assessments did vary between individuals within the sample due to differing number of chemotherapy cycles administered, chemotherapy cycle length, and treatment delays. The post-chemotherapy time point (T5) represented a period of 5–8 months from the baseline assessment. This variability has implications for nutritional outcome measures as weight gain has previously been shown to vary with duration of treatment in studies of women receiving adjuvant chemotherapy for breast cancer [29].

## Conclusions

Despite the stated limitations, this study characterised, for the first time, changes in taste perception and food hedonics over repeated chemotherapy cycles in women with early breast cancer and provided evidence that taste per se, as opposed to other elements of flavour is adversely affected at key points during chemotherapy. Findings will inform the design of future studies seeking to understand the mechanisms of changes in taste perception during chemotherapy, and ultimately, the design of interventions aimed at reducing the negative nutritional consequences of chemotherapy treatment. Understanding more about sensory risk factors for weight gain in breast cancer groups should be prioritised in future clinical research, given the link between obesity and poorer outcomes in this population.

### Implementation of findings into practice

Patients do not systematically receive specific information regarding the possible nature, timing of onset and duration of taste problems by health professionals, nor are possible consequences of changes or management strategies routinely discussed. This research has generated new evidence to guide assessment and predictors of chemotherapy induced sensory and hedonic changes. Findings of the study will shape tailored patient information provision in preparation for chemotherapy.
